# Aromatic Profiling of New Table Grape Varieties Using Gas Chromatography/Mass Spectrometry and Olfactometry

**DOI:** 10.3390/plants13131820

**Published:** 2024-07-02

**Authors:** Federica Bonello, Fabio Danieli, Vasiliki Ragkousi, Alessandra Ferrandino, Maurizio Petrozziello, Andriani Asproudi, Pierfederico La Notte, Costantino Silvio Pirolo, Vincenzo Roseti

**Affiliations:** 1 Council for Agricultural Research and Economics-Research Centre for Viticulture and Enology (CREA-VE), Via P. Micca 35, 14100 Asti, Italymaurizio.petrozziello@crea.gov.it (M.P.); andriani.asproudi@crea.gov.it (A.A.); 2 Department of Agricultural, Forest, and Food Sciences (DISAFA), University of Turin, Largo Paolo Braccini 2, 10095 Grugliasco, Italy; fabiodanieli1993@gmail.com (F.D.); alessandra.ferrandino@unito.it (A.F.); 3 CRSFA—Centro di Ricerca e Sperimentazione e Formazione in Agricoltura “Basile Caramia”, Via Cisternino, 281, 70010 Locorotondo, Italy; pierfederico.lanotte@ipsp.cnr.it (P.L.N.); vr.plants@gmail.com (V.R.); 4 Italian Variety Club, Via Cisternino, 281 c/o CRSFA Basile Caramia, 70015 Locorotondo, Italy; costantino.pirolo@gmail.com; 5 SINAGRI S.r.l.—Spin off of the University of Bari, Via G. Amendola, 165/A, 70126 Bari, Italy

**Keywords:** aromatic compounds, GC-MS, GC-O, table grape, flavor

## Abstract

The aim of this study is the aromatic characterization of new table grape varieties, namely Guzun (*V. vinifera*), Melona (*V. vinifera*), Cotton Candy (*V. vinifera*), IVC SA3 (*V. labrusca*), and IVC SB1 (*V. labrusca*). The qualitative and quantitative analysis of odorant molecules present in the berries allows for the definition of the aroma profile of the grape. This analysis benefits from the progress of analytical techniques and sensory methodologies. Gas chromatography/mass detection enable the efficient detection of the substances present and their concentrations. Through the coupling of gas chromatography with sensory detection (gas chromatography-olfactometry), it is possible to correlate the compounds detected by gas chromatography with olfactory stimuli, exploiting the human olfactory system. Aroma, a significant flavor component, is an important attribute of table grape that contributes to defining their quality. This characteristic is highly valued by consumers, and consequently, the market asks for table grapes with a particular or new aroma. Aromatic characterization is a crucial step in the study of the table grape varieties to evaluate their potential at the commercial level or, for instance, in breeding programs focusing on organoleptic properties.

## 1. Introduction

Traditionally grown in a few countries, table grapes are now cultivated in about 90 nations [1], making them a seasonally adjusted product available year-round, traded, and consumed globally [2]. Over the last 20 years, the sector has shown positive trends worldwide in terms of production (+70%), consumption (+73%), and international trade (+50%), attracting new producers and/or consumer countries [3,4]. In 2021, of the 69.6 million tons of wine, table and dried grape produced globally from a vineyard surface area of 7,327,311 ha, 43.3% (30.1 million tons) consisted of table grapes. China was the largest producer (36.2%), followed by India (11.9%), Turkey (6.2%), Egypt (4.9%), Iran (4.5%), Uzbekistan (4.1%), and Italy (3.3%) [5]. Italy is the leading table grape producer in the EU, with more than one million tons produced commercially in 2021 from 47,019 ha. This production is distributed across several regions but is concentrated in Apulia (24,685 ha) and Sicily (18,720 ha), which together represent more than 93% of the national production [4,6].

The table grape is characterized by several attributes related to visual characteristics and physico-chemical properties that form the basis for evaluating its quality and consumer acceptability. These attributes include attractive appearance, fabric integrity, being free of decay, freshness, juiciness, sweetness, and texture characteristics. Texture is defined by mechanical parameters such as skin thickness, friability and consistency, pulp firmness, compactness, crispness, or crunchiness (which is the most desirable attribute), as well as the hardness, cohesiveness, gumminess, springiness, chewiness, and resilience of the whole berry. Other important attributes are color, size, shape, flavor, and aroma [7,8,9,10,11,12,13,14,15].

The aroma of the grape berries is determined by odorant compounds located in the pulp and primarily in the skin. These compounds exist in both free-odor-active and bound-odorless forms as glycosides and amino acid conjugates [16]. The free volatile forms, released under cell wall rupture conditions, contribute directly to the aroma, whereas the bound non-volatile forms require further modifications to be perceived [16,17]. Given that most odorous compounds normally accumulate in the latter form, constituting the aroma precursors or the potential aroma, they are not detected during berry consumption. Consequently, most grape varieties appear neutral in aroma or are not inherently odorous [10,16]. Grape berry volatiles belong to several molecular classes, such as aldehydes, alcohols, esters, monoterpenes, sesquiterpenes, norisoprenoids, lactones, ketones, polyfunctional thiols, and methoxypyrazines, synthesized through different metabolic pathways [16,18]. These molecules are plant secondary metabolites produced constitutively or in response to various stimuli. They are essential for the plant’s life, playing crucial roles in interactions with biotic and abiotic factors, participating in defense mechanisms, and facilitating plant-to-plant communications [19]. 

Having a strong impact on buying decisions, the food aroma is of significant interest for academics and industry professionals. The qualitative–quantitative analysis of grape aroma compounds, as well as the knowledge of their origin and evolution, are fundamental for understanding how to modulate odorant concentrations and profiles and ultimately consumer perception.

In this work, we present the results obtained by analyzing the aroma of new table grape varieties through gas chromatography/mass detection, and olfactometric analysis. This comparison between olfactometry and gas chromatographic analysis of new table grape varieties constitutes a relatively underexplored aspect and represents a significant novelty. This study offers valuable insights for both the scientific community and the production industry, guiding future research endeavors and enhancing cultivation and selection practices within the table grape sector.

## 2. Materials and Methods

The research was performed on five table grape varieties, harvested during 2022, provided by the Italian Variety Club (www.reteivc.it, accessed on 1 April 2024), a network of 23 private companies that joined in 2014 to start an extensive table grape breeding program in Southern Italy. The varieties are Guzun (*V. vinifera*), Melona (*V. vinifera*), Cotton Candy (*V. vinifera*), IVC SA3 (*V. labrusca*), and IVC SB1 (*V. labrusca*). The aromatic compounds were first isolated using SPE (Solid Phase Extraction) and subsequently analyzed using GC-MS (Gas Chromatography-Mass Spectrometry) and GC-O (Gas Chromatography-Olfactometry).

### 2.1. Sample Preparation and Extraction of Volatile Compounds

The sample was prepared as follows: the berries of each cultivar, with the peduncles attached to minimize oxidation, were removed from the clusters. The grapes utilized were carefully chosen through a randomized selection process conducted in the field. This ensured that the selection was not biased, and each grape had an equal chance of being included. Three batches of every cultivar are prepared of the same weight and immediately frozen in plastic bags. 

The grapes were partially thawed, facilitating the removal of pedicels and the elimination of seeds by cutting each single berry in two halves. The pulps together with skins, with 0.15 g of sodium metabisulphite (Sigma-Aldrich, St. Louis, MO, USA) to exploit its antimicrobial and antioxidant properties, were homogenized for 1 min (Braun, Frankfurt, Germany, N2820–19737) and centrifuged at 4000 rpm for 10 min at 15 °C (Eppendorf 5810R, Hamburg, Germany). The solid residue was diluted with tartaric acid buffer at pH 3.1 (Tartaric acid 1 M, tartrate 1.2589 M) and subjected to a second centrifuge. The supernatants were transferred into a flask, to which an enzymatic preparate of pectinases was added as a mix of polygalacturonase, pectin lyase, and pectin esterase (Lallzyme HC™, Lallemand, Montreal, QC, Canada) to improve the release of aroma compounds, brought up to volume with the tartaric acid buffer, and placed in the fridge at 8 °C for 24 h. This acidic buffer is an optimum solvent, allowing the extraction of a high number and amount of free and glycosylated volatile compounds [20].

The following day, the sample was filtered on cellulose paper (589/1, Schleicher & Schuell, Dassel, Germany) to eliminate particulate matter. It was then diluted in a 1:1 ratio with deionized water and spiked with a known concentration of 1-heptanol as an internal standard. The solution was loaded on a 5 g C18 cartridge (Isolute^®^, Biotage, Uppsala, Sweden) previously conditioned with 20 mL of methanol (412383, Carlo Erba, Milan, Italy) and equilibrated with 50 mL of water. The cartridge was washed with 50 mL of water, and, once dried, the free volatile compounds retained were eluted with 30 mL of dichloromethane (463342, Carlo Erba, Milan, Italy). The extract was water-dried with sodium sulfate anhydrous (483005, Carlo Erba, Milan, Italy), concentrated under a gentle nitrogen stream, placed in a vial, and frozen.

### 2.2. Analysis of Volatile Compounds by GC-MS

Separation, identification, and semi-quantification of volatile compounds were performed through GC-MS analysis. 

Prior to injection, the extract was concentrated under a stream of pure nitrogen. A total of 1 μL was injected into the gas chromatograph (7890A, Agilent, Santa Clara, CA, USA) equipped with a Zebron™ ZB-WAX capillary column, length 60 m, inner diameter of 0.25 mm, film thickness of 0.25 μm, (Phenomenex, Torrence, CA, USA), and coupled to a quadrupole mass spectrometer with triple-axis detector (5975C, Agilent). Injection was in splitless mode (2 min splitless time). The temperatures in the injector port and the transfer line were maintained at 250 and 230 °C, respectively. Helium was used as a carrier gas at a constant flow rate of 1 mL/min. The oven temperature program was set as follows: 3 °C/min from 40 to 60 °C and held for 2 min; 2 °C/min to 190 °C; 5 °C/min to 230 °C and held for 15 min. Electron impact was set at 70 eV, and the acquisitions were performed in full-scan mode.

The identification of aroma compounds was conducted by comparing the mass spectra with those of authentic standards, where available. Additionally, identification of volatile compounds was performed using the NIST14 and WILEY275 databases, with data processing and analysis conducted through the Agilent ChemStation software (D.02.00 version). A match quality, defined as a percentage measure of the correspondence between the unknown mass spectrum and the reference spectrum in the library greater than 90%, was considered reliable.

The linear retention index (LRI) was also employed for compound identification. The LRI was calculated by comparing the retention times of the compounds to a homologous series of n-alkanes (n6–n40) and then comparing the obtained values with those reported in the literature). These data are reported in Table 1, along with the IUPAC nomenclature of each mentioned compound and its corresponding odor, as found in the bibliography. Compounds were semi-quantified as internal standard equivalents (i.e., by relating the peak area of the analytes to the peak area of the internal standard), using 1-heptanol for free volatiles. All analyses were performed in triplicate.

### 2.3. Analysis of Volatile Compounds by GC-O

The concentrated extract (1 μL) of free volatile compounds from each cultivar was subsequently injected into the GC-O system. This was a GC (2010-Plus, Shimadzu, Kyoto, Japan) equipped with a flame ionization detector (FID), equipped with a Stabilwax^®^-DA capillary column (30 m × 0.25 mm × 0.25 μm) (Restek, Bellefonte, PA, USA), and coupled with a PHASER sniffing port (GL Sciences, Tokyo, Japan). The injection was in splitless mode (2 min splitless time). The method used was the one described by Campo et al. [24] and partially adapted as detailed below.

Detector and injector temperatures were maintained at 250 °C. Helium was used as a carrier gas at a constant flow rate of 2 mL/min. The oven temperature program was set as follows: 40 °C for 1 min; 10 °C/min to 45 °C and held for 1 min; 2 °C/min to 80 °C; 6 °C/min to 230 °C and held for 4 min. The total separation time was 49 min. The sniffing port was equipped with a humidifier filled with deionized water. The transfer line, through which the effluent from the capillary column reaches the olfactometric port, was heated, and the temperature was controlled manually starting at 60 °C for 10 min, then 80 °C for 10 min, 140 °C for 10 min, 200 °C for 10 min and finally held at 250 °C for 9 min.

The olfactometry signal was obtained by using a panel of 6 judges composed of 4 women and 2 men, aged between 24 and 58 years (average = 43 years). The sniffers annotated the time, odor description, and odor intensity (1 = very weak, 2 = weak, 3 = clear, 4 = strong, 5 = extremely strong) when they detected an aroma.

Compound identification was performed by comparing odor descriptors from the literature and chromatographic retention indices, calculated in the same manner as previously described for GC-MS analysis. The calculated LRI, literature-derived LRI, and olfactory recognition of substances are comprehensively presented in Table 1. The data obtained from the GC-O screening were processed using a strategy classified as the direct intensity method. To determine the most important odorous compounds present in the sample, for each detected odorant, a GC-O score was defined through the calculation of the modified frequency in percentage (% MF), using the formula proposed by [25]:

$$MF\left(\%\right)=\sqrt{F\left(\%\right)\times I(\%)}$$

where F (%) is the detection frequency of an aromatic attribute expressed as a percentage of the total number of judges on the panel, and I (%) is the average intensity expressed as a percentage of the maximum intensity [26]. In other words, MF (%) measures the olfactory intensity of a compound, taking into consideration its detection by the assessors. The odorous stimuli detected with a MF (%) higher than 40 were considered the most important compounds present in each sample, while the odorants not reaching the aforementioned value in any of the studied samples were evaluated as noise and eliminated. This choice is arbitrary, but nevertheless, it is very useful.

This technique, when carried out with expert evaluators in order to obtain fast, repeatable, and generally consistent results even in a single run, ensures excellent results [27,28].

## 3. Results

The analysis showed remarkable differences in the profile and concentration of volatile compounds among the table grape varieties considered. In order to provide a comprehensive understanding, the results and observations will be presented by analyzing each variety separately, focusing on their distinct aromatic characteristics.

### 3.1. The Aromatic Profile of Guzun Grapes

The Guzun grape berries are large and have a shape reminiscent of an amphora. The seeds, numerous and of considerable size, were well lignified. During the sample preparation phase, terpene compounds were clearly perceptible, leading to the assumption that the grape was of the Muscat type. The GC-MS results of Guzun, reported in Table 2, confirmed that this table grape variety is characterized by significant concentrations of varietal compounds, namely terpenes.

In particular, the analysis of the free volatile compound fractions has highlighted the presence of linalool, HO-trienol, α-terpineol, citronellol, geraniol, (*Z*)-linalool oxide, (*E*)-linalool oxide, epoxylinalool, citral, and geranic acid that constitute the monoterpene profile. In addition, considering the linalool/geraniol ratio, it is possible to observe a similarity between the Guzun and the Malvasia (a group of wine grape varieties) as opposed to the Muscat variety family, despite the lower terpene content.

An elevated quantity of ethyl esters was detected, namely ethyl butyrate, ethyl octanoate, ethyl decanoate, ethyl hexadecanoate, and ethyl dodecanoate. Typically, these molecules are not found in the *V. vinifera* grape due to the very low activity of AAT (Alcohol Acyltransferase) enzymes during ripening. These esters enrich the floral and fruity bouquet of the grape. The C6-compounds, (*E*)-2-hexenal, hexanol, and (*E*)-2-hexenol, characterized by negative herbaceous notes, were released during the skin shredding, and the same could happen during grape consumption.

The GC-O analysis provided some information about the olfactory importance of the single volatile molecules present in the berries. For each detected odorant, the modified frequency in percentage (% MF) was defined as the GC-O score. This score was calculated as the geometric mean of the detection frequency of an aromatic attribute and the intensity of the olfactory stimuli, both expressed as percentages. The GC-O results for Guzun grapes are reported in Table 3. The analysis identified 45 compounds, of which more than 40 were detected, and 21 of these had an MF (%) greater than 60: eugenol, 2-phenylethanol, geraniol, phenylacetic acid, furaneol, vanillin, 4-ethylguaiacol, nonanoic acid, hexanol, isovaleric acid, 5-ethoxydihydro-2(3H)-furanone, guaiacol, 2-methoxy-3-sec-butyl pyrazine, linalool, 3-mercaptohexyl acetate, rotundone, 4-mercapto-4-methyl-2-pentanone, octanoic acid, methoxybenzaldehyde, sotolon, and decanoic acid.

Among these compounds, specifically considering the monoterpenes, linalool was perceived as characterized by orange blossom, floral, rose, and white flower odors, while geraniol was noted for its rose, lavender, lemon, and fruity scents.

In addition to most of the molecules detected through the GC-MS, the olfactometric analysis of Guzun samples enabled the identification of some key odorants known as polyfunctional thiol compounds. These molecules are hardly detected by GC-qMS due to their presence at trace levels, and their determination requires specific analysis [30]. Polyfunctional thiols or volatile thiols have a very low perception threshold (measured in nanograms per liter). The ability of assessors to detect these compounds demonstrates their remarkable sensitivity to the human nose. These compounds usually accumulate in their non-volatile form as aminoacid conjugates, bound to S-cysteine or S-glutathione, and the release of odor-active volatiles requires the activity of microbial enzymes (carbon-sulfur lyases) [31]. However, the boxwood odor, associated with 3-mercaptohexyl acetate and 4-mercapto-4-methyl-2-pentanone, has been found in the free volatile fraction, and it was already clearly perceptible during the sample preparation phase.

Like the thiol compounds, 2-methoxy-3-sec-butylpyrazine, 2-ethyl-3-methylpyrazine, and 2-acetyl-3-methylpyrazine were exclusively detected by olfactometric analysis. The pyrazines are usually present in low concentration but are potent odorants with a very low perception threshold. The panel identified 2-methoxy-3-sec-butyl pyrazine and 2-ethyl-3-methylpyrazine as having green pepper and pea odors, while 2-acetyl-3-methylpyrazine has cucumber and tzatziki scents.

Briefly, in the Guzun variety, the aroma of the grape is predominantly influenced by the complex interplay of various compounds, including terpenes such as linalool and geraniol, specific thiol compounds known as polyfunctional thiols, and distinct pyrazines. These elements together contribute to a rich bouquet of scents ranging from floral notes to more robust odors like green pepper and cucumber, demonstrating the multifaceted nature of the Guzun grape’s aroma.

### 3.2. The Aromatic Profile of Melona Grapes

The berries of the Melona grape presented a medium size with a globular form. The seeds, medium in size, were not lignified. During the sample preparation phase, an intense odor of melon was emitted from the berries.

The GC-MS results, as shown in Table 2, led to the classification of the Melona table grape as a variety characterized by a neutral aroma.

The monoterpenes in the free volatile compound fractions are presented in very low concentrations and numbers. The prevalence of ester and ketonic notes is accompanied by caramelized and fruity aromas. In particular, the high concentration of (*E*)-cinnamic acid imparts sweet spice and caramel scents to the grape. Furthermore, the combination of esters such as ethyl butyrate, ethyl decanoate, ethyl hexadecanoate, and ethyl dodecanoate results in olfactory notes of tropical fruits, in particular melon, which become distinctly evident upon tasting the grape.

The GC-O results of the Melona grape are reported in Table 4, indicating twenty-three compounds with a MF (%) value higher than 60. However, o these are mainly molecules like furaneol, sotolon, eugenol, phenylacetic acid, and acetophenone, which, although at the end of the analysis they present the highest MF (%) value, give odors like pastry, cookies, and honey.

These analytical evaluations confirm the grape’s neutral aroma profile.

Of note, the panel identified a melon odor associated with the compound 2-decenal. This molecule can contribute to the characteristic aroma from which the grape takes its name. Considering the compound odor descriptors reported in the literature, only 2,4-heptadienal was indicated as possibly possessing a melon scent, but the assessors perceived it as having a cucumber and bug-like odor [28,29]. While the literature suggests that only 2,4-heptadienal may have a potential melon scent, the assessors perceived it as more similar to cucumber and having a bug-like odor. The melon aroma is likely a result of a combination of molecules, including specific esters.

### 3.3. The Aromatic Profile of Cotton Candy Grapes

The Cotton Candy variety was initially selected in 2003 by David Cain in California through the cross-pollination of a dozen Californian varieties. Several years passed from this moment until its placement in the retail stores. The popularity of this grape increased over time thanks to the distinctive candy floss and sweet odors, coupled with the seedless character of the berries. Nowadays, it is cultivated in 13 countries, with a production of more than 34,000 tons destined for the main markets. In Italy, some licensed producers in Apulia cultivate this variety, with production primarily aimed at Germany and Switzerland. The grape finds great appreciation from the consumer, and the demand is constantly growing. This is an example of the commercial importance of the aroma attribute of table grapes [32,33].

The Cotton Candy berries presented a high size with an oblong shape. After tasting the berry, the sweet and candy notes were perceived. The GC-MS results are reported in Table 2.

This variety presents a notably low content of monoterpenes as free volatiles. However, it is characterized by the presence of eucalyptol, which was also detected by the panel in the olfactometric analysis (Table 5) as balsamic, mint, and sage scents [28,29]. Unlike the other varieties analyzed, the extremely high amounts of 3(2H)-furanone, furaneol, and γ-butyrolactone confer the typical notes of caramel, candyfloss, and pastry. In addition, furaneol is also often perceived as a strawberry and candy odor descriptor. Benzaldehyde, with its almond and cherry scent, also contributes to the aromatic complexity of the variety.

Summarizing these results, the Cotton Candy variety is evaluated as neutral in the aroma character, as noticed for the Melona grape.

In the olfactometric analysis, 23 compounds that exceeded a threshold of 60 MF%. were identified. However, these molecules mainly contribute caramelized (furaneol, phenylacetic acid, vanillin) and spicy notes (sotolon, eugenol, 4-vinylphenol, rotundone, 4-ethylguaiacol, methoxybenzaldehyde, and ethyl hydrocinnamate). Therefore, it confirms the intense scent of candyfloss and the candy aromas typical of this grape.

With acute intensity, the rotundone was perceived with its characteristic pepper odor, a molecule that can also be distinguished in the other grapes. This compound is present at a trace level and, like the thiols and the pyrazines, is hardly detected by GC-MS analysis. Through the sensitivity of the human nose, it was possible to perceive it and then consider rotundone as one of the key fragrances of the grape.

Despite its typical candyfloss, candy, and sweet odors, the Cotton Candy variety is overall considered neutral in its aroma.

### 3.4. The Aromatic Profile of IVC SB1 Grapes

The berries of the IVC SB1 grape presented a small size with a globular shape. The seeds are absent.

The GC-MS results are reported in Table 2. In the free volatile compound fractions, esters predominate the aromatic profile in *V. Labrusca*. Ethyl butyrate, ethyl decanoate, and ethyl hexadecanoate are present in very high concentrations. They exhibit the typical fruity notes reminiscent of the ripe strawberry flavor [34].

Compared to the other varieties analyzed, elevated quantities of (*E*)-2-hexenal, hexanol, and (*E*)-2-hexenol can give fruity and cut grass scents.

The monoterpenes are present in very low concentrations. The presence of thymol is characteristic, a monoterpenic phenol with spicy, thyme, and medicinal odors.

The GC-O results (Table 6) highlighted 14 molecules with a modified frequency higher than 60%.

Without consideration of the significant geraniol contribution, perceived by the panel as rose, citrus, floral, and white flower scents, the most important compounds for the aroma characterization of the IVC SB1 grape are furaneol (smoky, candyfloss), ethyl hydrocinnamate (cinnamon, spicy), maltol (cinnamon, spicy, cooked), and eugenol (spicy, cloves, vanilla).

Prominent in the results are also the caramelized (maltol, phenylacetic acid, lauric acid) and floral (e.g., 2-phenylethanol) notes.

The grape bouquet is therefore complex, although it may not prominently showcase distinct varietal aromas.

### 3.5. The Aromatic Profile of IVC SA3 Grapes

The IVC SA3 berries presented a medium-high size with an oblong shape. The grapes are seedless. During the sample preparation phase, fruity and candy odors were perceptible.

The aromatic peculiarity of the IVC SA3 variety clearly emerges from GC-MS analysis (Table 2).

Among the free volatile compounds, especially when compared with the other varieties examined, the terpene content is very high. The monoterpene profile is formed mainly by linalool, HO-trienol, citronellol, geraniol, linalool oxides, and citral. In particular, linalool and, above all, geraniol are abundantly present. All these molecules, both individually and synergistically, contribute to defining the floral and fruity aroma, characterized, for instance, by rose, orange blossom, and citrus notes.

The monoterpene level and ratio between linalool and geraniol place this variety among varieties like Brachetto and Malvasia, but also among Gewürztraminer and Moscato rosa, as found in [35,36,37].

Unlike the IVC SB1 variety, in the IVC SA3 grape, the ester profile is not determinant. However, some ethyl esters are present in not negligible concentrations and can confer their typical fruity notes.

The olfactometric analysis of the IVC SA3 grape (Table 7) highlights 14 molecules with a MF (%) higher than 60%. Among these, the rotundone (spicy black pepper) presents the highest GC-O score, a very interesting result for the varietal characterization of a cultivar with the blood of *V. labrusca*. A modified frequency of 83.7% indicates that this molecule is present in considerable amounts, even if it was not possible to detect the rotundone via GC-MS analysis.

Furaneol (cookies, candyfloss, caramel), eugenol (cloves, spicy, phenolic), sotolon (curry, liquorice), and vanillin (vanilla) resulted in a modified frequency of 77.5%.

Following, stand out the monoterpene linalool (orange blossom, floral, rose, white flower), geraniol (lemon, rose, floral), citral (mint, lemon), and citronellol (rose, peach, dried fruit).

The analysis of the aroma of IVC SA3 points out the high aromatic complexity of this variety. Its bouquet includes all types of odors, ranging from sweet, pastry, and caramel notes to spicy fragrances and fruity and floral scents.

## 4. Discussion

The analysis conducted on the five table grape varieties has revealed substantial differences in the profile and concentration of the odorous molecules. At the same time, each cultivar presents peculiarities that render its aroma unique, as is typical in grapevine.

**Figure 1:**
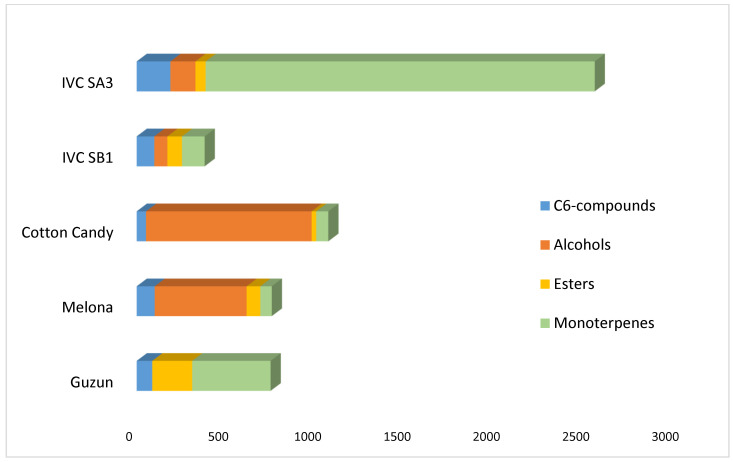
Graphic representation of the sum of the concentrations (μg/kg), calculated from the media of the triplicates of the free volatile compound fractions, of the most important C6-compounds, alcohols, esters, and monoterpenes of the five varieties analyzed.

The histogram reported in Figure 1 provides an overall view of the most important odorants, merged by molecular classes, as determined by the GC-MS analysis of the free volatile compound fractions.

The total free monoterpene concentration (2173 μg/kg) can place the IVC SA3 variety into the non-Muscat aromatic cultivars (1–4 mg/L), according to the classification usually used for the wine grape [35,36,37]. Considering both the GC-MS and the GC-O analyses, among the five varieties analyzed, the IVC SA3 turned out to be the grape with the highest aromatic richness. Its bouquet exhibits a high level of complexity and diversity, including all typologies of odors.

Beyond the IVC SA3, the Guzun grape is characterized by a notably rich terpenic profile, even if the total monotepene concentration (437 μg/kg) is lower. The high ester content (223 μg/kg) gives this variety intermediate features between *V. labrusca* and *V. vinifera*. The olfactometric analysis also highlights the contribution of thiol and pyrazine compounds to the aroma. Overall, the Guzun grape is characterized by varietal compounds of high quality.

The IVC SB1 grape presents the classic aroma profile of the *V. labrusca* varieties, with a low monoterpene content (126.51 μg/kg) and a substantial amount of esters (81.34 μg/kg). The olfactometric analysis pointed out the moderate complexity of the grape bouquet, even if not too varietal.

The Cotton Candy grape is characterized by a higher content of alcohols (924.35 μg/kg) with a prevalence of 2-phenylethanol, conferring a strong rose scent. The typically reported candyfloss, candy, and sweet odors were confirmed by the analysis through the high level of compounds like furaneol. Overall, the Cotton Candy variety is considered neutral in its aroma.

The Melona grape contains a high amount of alcohol (513.15 μg/kg) and a noteworthy level of esters (76.42 μg/kg), in particular the ethyl hexadecanoate with distinct fruity notes. This variety is considered neutral in aroma and the least interesting among the grapes analyzed.

## 5. Conclusions

The grapevine and its berries produce an impressive number of volatile molecules. More than 800 aromatic compounds have been detected in grapes [38]. Each grape is characterized by a unique aroma profile shaped, based on genotype, by complex physiological and biochemical events that regulate plant growth and development and responses to stressful conditions. The grape variety genotype is by far the principal factor in determining the production of volatile compounds. Aroma is a complex quantitative trait controlled by multiple genes [39,40,41]. Understanding the origin and regulation of odorant molecules is crucial for improving viticulture and assessing the impact of climate and seasons.

In recent years, grapes with specific flavors have gained significant appreciation in the market [42], since flavor and aroma are among the most crucial attributes influencing consumer acceptance and preferences for table grapes [29,41,43,44,45,46,47,48]. Since odor input processing involves the amygdala recalling emotional behavior, aroma also has a hedonic valence, and the pleasantry derived from food sensory perception enriches our lives [46,49,50]. Investigating the mechanisms and impact of external factors and physiological parameters on aroma perception is crucial for defining and modulating the sensory processing patterns of consumers.

The huge progress of analytical techniques and sensory methodologies has facilitated deep studies on the composition and concentrations of grape odorant compounds [16], opening a wide range of applied uses, from the study of consumer preferences to the development of new products such as new varieties obtained with genetic improvement. The development of new varieties, exploiting the enormous *Vitis* genetic diversity, is the principal way to meet the market demand for aroma-rich table grapes [16,41,48].

## Figures and Tables

**Table 1:** ^1^ LRI Lit.: Linear Retention Index values retrieved from the literature. ^2^ LRI Cal.: Linear Retention Index calculated comparing retention times of a homologous series of n-alkanes and analytes, separated with the used GC method. ^3^ Ref.: The references from which LRI values were obtained from the literature are listed below. ^4^ Gr.: Compounds are grouped according to their chemical structure. C6 alcohols with a six-carbon skeleton (also known as leaf alcohols), A: alcohols; AC: acids; AL: aldehydes; B: benzenoids; EE: esters; K: ketones; S: sesquiterpenes; T: terpenes; (a) [21], (b) [22], (c) [23].

LRI Lit. ^1^	LRI Cal. ^2^	Ref. ^3^	Gr. ^4^	Name	IUPAC Name	Odour Descriptor
1196–1238	1233	a	C6	*trans*-2-hexenal	(*E*)-hex-2-enal	Grass, green, herbaceous, leaf, fruity, spicy
1316–1377	1359	a	C6	1-hexanol	hexan-1-ol	Green, cut grass, herbaceous, flower, fruity, etherial, oil
1377–1419	1413	a	C6	*trans*-2-hexen-1-ol	(*E*)-hex-2-en-1-ol	Herbaceous, green, leaf, herbal, walnut, fruity,
1198–1217	1210	a	A	isoamyl alcohol	3-methylbutan-1-ol	Foot, solvent, fusel, alcoholic, pungent, etherial, cognac, brandy, wine, fruity, banana, molasses
1289–1339	1302	c	AL	2-Heptenal	2-Heptenal	Green, fatty
1630–1655	1643	nist	AL	*trans*-2-decenal	(*E*)-dec-2-enal	Orange, floral, rose, green
1719	1715	nist	AL	2,4-decadienal	(*2E,4E*)-deca-2,4-dienal	Aldehyde, fatty, oily, seaweed, sweet, citrus, green
1824	1800	nist	AL	Tridecenal	(*E*)-2-Tridecenal	Waxy, citrus rind, tangerine, fatty, creamy, soapy, cilantro
1037	955	a	EE	Ethyl butyrate	Ethyl butyrate	Fruity, strawberry, apple, sweet, lactic
1428–1441	1436	a	EE	Ethyl octanoate	Ethyl octanoate	Sweet, floral, fruity, banana, pear, pineapple, brandy, fat, waxy, musty, creamy, dairy
1626–1644	1636	a	EE	Ethyl decanoate	Ethyl decanoate	Fruity, grape, apple, nutty, sweet, waxy, oily, wine, yeast
2250	2250	a	EE	ethyl palmitate	Ethyl Hexadecanoate	Waxy, fruity, creamy, milky, balsamic
1826–1850	1841	a	EE	ethyl laurate	ethyl dodecanoate	Sweet, waxy, soapy, creamy, floral, fatty, fruity
1727–1809	1782	a	B	methyl salicylate	methyl 2-hydroxybenzoate	Peppermint, green, sweet, phenolic, camphoreous
1636–1697	1684	c	AC	Isovaleric acid	3-methylbutanoic acid	Cheese, sweat
1807–1873	1858	a	C6	hexanoic acid	hexanoic acid	Rancid, cheese, fatty, sweat
1922–2002	1963	nist	AC	*trans*-2-Hexenoic acid	(*E*)-2-Hexenoic acid	Must, fat, cheesy, caramel
1922–1945	1898	nist	AC	*cis*-2-Hexenoic acid	(*Z*)-2-Hexenoic acid	
2051	2059	c	AC	Octanoic acid	Octanoic acid	Rancid, perspiration, plastic, cheese
2152	2170	c	AC	Nonanoic acid	Nonanoic acid	Nutty, green, fat, waxy, cheesy, dairy
2258	2272	c	AC	Decanoic acid	Decanoic acid	Rancid, perspiration, fat
2503	2479	c	AC	Lauric Acid	Dodecanoic acid	Metal
2886–2946	2876	c	AC	Palmitic acid	n-Hexadecanoic acid	Waxy, creamy, candle
2957	2687	nist	AC	Palmitoleic acid	9-Hexadecenoic acid	
3157	3098	nist	AC	Linoleic acid	Octadienoic acid	
3157–3200		nist	AC	Oleic acid	9-Octadecenoic acid	Fat
1507–1564	1553	a	T	linalool	3,7-dimethylocta-1,6-dien-3-ol	Citrus, orange, grape, floral, lavender, rose, waxy
1580–1616	1619	a	T	OH trienol	3,7-Dimethyl-1,5,7-octatrien-3-ol	Fresh, floral, hyacinth, fruity
1659–1724	1702	a	T	α-terpineol	2-[4-methylcyclohex-3-en-1-yl]propan-2-ol	Floral, lilac, sweet, anise, mint, green, oil
1734–1789	1775	a	T	citronellol	3,7-dimethyloct-6-en-1-ol	Floral, rose, sweet, citrus, green, fatty
1795–1865	1857	a	T	geraniol	(2*E*)-3,7-dimethylocta-2,6-dien-1-ol	Citric, orange, floral, roses, geranium, citronella, fruity
1410–1478	1448	a	T	*cis*-furan linalool oxide	2-(5-ethenyl-5-methyloxolan-2-yl)propan-2-ol	Floral, green
1429–1481	1476	a	T	*trans*-furan-linalool oxide	2-(5-ethenyl-5-methyloxolan-2-yl)propan-2-ol	Woody, floral
1698–1778	1746	b	T	*trans*-pyran Linalool oxide	(3R,6S)-6-ethenyl-2,2,6-trimethyloxan-3-ol	
1641–1706	1727	nist	T	citral	2,6-Octadienal, 3,7-dimethyl-	Lemon, lemon peel, juicy, green
2130–2198	2193	nist	T	Thymol	5-methyl-2-propan-2-ylphenol	Spicy, phenolic, chemical, medicinal, thyme
1211	1289	nist	T	Eucalyptol	1,3,3-Trimethyl-2-oxabicyclo[2.2.2]octane	Camphoric, mint, sweet, eucalyptus, camphoraceous
2347	2357	c	T	geranic acid	(2*E*)-3,7-dimethylocta-2,6-dienoic acid	Green, woody, sweet
2340–2370	2356	nist	S	Farnesol	2,6,10-Trimethyl-2,6,10-dodecatrien-12-ol	Green, floral, Lily
1250–1314	1309	nist	K	Acetoin	3-hydroxy-2-butanone	Lactic, fatty, butter, cream, dairy, milky
1667	1665	a	K	Acetophenone	Ethanone, 1-phenyl	Must, flower, spicy, almond, nuts, fruity, cherry, strawberry
2184		nist	K	Methoxyacetophenone	1-(2-methoxyphenyl)ethanone	
1648	1632	a	K	γ-Butyrolactone	2(3H)-Furanone, dihydro	Caramel, sweet, creamy, oily, fatty
1481–1555	1532	a	B	benzaldehyde	benzaldehyde	Sweet, fruity, roasted, almond, nutty, fragrant, burnt sugar, powdery, cherry, floral
1662	1630	c	B	Phenylacetaldehyde	Benzenacetaldehyde	Flowery, rose, green, honey, powdery, chocolate, earthy
1859–1944	1923	a	B	2-phenylethanol	2-phenylethanol	Sweet, floral, rose, bready, honey, aromatic
1821–1905	1888	a	B	benzyl alcohol	phenylmethanol	
2832–2844		nist	B	(*E*)-Cinnamic acid	(*Z*)-3-Phenyl-2-propenoic acid	
2448	2294	c	B	Benzoic acid	Benzoic acid	Urine
2357–2420	2415	b	B	4-vinylphenol	4-ethenylphenol	Almond shell, chemical, phenolic, medicinal, musty
2531–2605	2590	b	B	vanillin	4-hydroxy-3-methoxybenzaldehyde	Vanilla, custard, sweet, creamy, phenolic
2578	2391	nist	B	Phenylacetic acid	Benzeneacetic acid	Honey, flower, rose, chocolate, tobacco, powdery, animal
3099	2880	b	B	homovanillic alcohol	2-(4-hydroxy-3-methoxyphenyl)acetic acid	
1993–2066	1981	c	K	Furaneol	2,5-Dimethyl-4-hydroxy-3(2H)-furanone	Candy, sweet, candyfloss, caramellic

**Table 2:** Concentrations (μg/kg) of the free volatile compound fractions in the grapes measured by GC-MS are provided. The content of the molecules is expressed as relative areas compared to 1-heptanol. Results are expressed as mean ± standard error. The mean (µ) and standard error (SE) are calculated from triplicate measurements. nd: not detected.

Compounds	Guzun	Melona	Cotton Candy	IVC SB1	IVC SA3
(*E*)-2-Hexenal	28.3 ± 3	19.6 ± 0.6	4.2 ± 0.3	75.5 ± 20.3	5.3 ± 0.7
Hexanol	23.7 ± 0.8	62.8 ± 8	18.2 ± 1.2	15.1 ± 1.1	145.3 ± 1.6
(*E*)-2-Hexenol	34.2 ± 8.6	18.4 ± 1	30.2 ± 1.9	7.8 ± 1.9	37.5 ± 0.8
Isoamyl alcohol	0.2 ± 0	1.2 ± 0	0 ± 0	0.1 ± 0	0.2 ± 0.1
2-Heptenal	19.7 ± 0.3	nd	15.5 ± 6.5	nd	5.2 ± 1
2-Decenal	nd	nd	8.2 ± 0.3	116.7 ± 4.1	1.4 ± 0.5
2,4-Decadienal	nd	nd	8 ± 0.6	nd	nd
(*E*)-2-Tridecenal	5.5 ± 0.3	3.1 ± 0.6	2.9 ± 0.1	nd	nd
Ethyl butyrate	6.5 ± 1	8.1 ± 2.3	2.3 ± 0.2	31.1 ± 2.5	16.5 ± 1.8
Ethyl octanoate	19.8 ± 1.2	nd	nd	nd	nd
Ethyl decanoate	151.2 ± 23.6	2.8 ± 0.2	5.1 ± 0.4	33.5 ± 2.5	7 ± 0.9
Ethyl hexadecanoate	6.1 ± 0.1	28.4 ± 1.1	6.1 ± 1.3	16.8 ± 1.3	33.4 ± 10.8
Ethyl dodecanoate	15.8 ± 0.9	34.6 ± 1.3	nd	nd	nd
Methyl salicylate	nd	2.6 ± 0.8	11.6 ± 2.3	nd	nd
Methylbutyric acid	nd	36.3 ± 1.1	3.9 ± 1	25.7 ± 1.2	8.2 ± 2.9
Hexanoic acid	nd	0.1 ± 0	0.1 ± 0	0.2 ± 0	0,2 ± 0
(*E*)-2-Hexenoic acid	58.3 ± 12.6	28.1 ± 1.5	38.6 ± 2	31.6 ± 2.3	48.6 ± 2.5
(*Z*)-2-Hexenoic acid	45.6 ± 2.3	22.6 ± 6.7	38.6 ± 2	nd	48.6 ± 2.5
Octanoic acid	3.9 ± 2.4	14.6 ± 2.8	15.9 ± 3.8	58.9 ± 2.7	18.1 ± 2
Nonanoic acid	11.1 ± 0.7	15.3 ± 1.6	15.7 ± 3.4	16.5 ± 4.5	17 ± 1.3
Decanoic acid	10.2 ± 2.8	23.9 ± 3.8	20.8 ± 1.4	27.2 ± 1	12.1 ± 1.1
Lauric Acid	47.2 ± 7.5	61.5 ± 18	15 ± 1.3	32.6 ± 6.1	26.3 ± 2.5
Palmitic acid	1643.2 ± 297.7	2615.7 ± 33.8	32.3 ± 2.3	1562.7 ± 203.7	4691.1 ± 510.5
Palmitoleic acid	83.4 ± 12.2	169.6 ± 65.8	nd	97 ± 2.1	118.7 ± 27.6
Octadienoic acid	45.2 ± 15.9	nd	91.4 ± 46.6	nd	69.3 ± 8.9
Oleic acid	nd	nd	63.4 ± 31.1	nd	136.7 ± 70.2
Linalool	41 ± 2.8	0.2 ± 0	1.2 ± 0.1	7 ± 0.6	310.2 ± 9.7
Hotrienol	2.5 ± 0.8	nd	nd	nd	29.8 ± 7.7
α-Terpineol	83.5 ± 4.9	46.1 ± 1.6	nd	nd	92.9 ± 18
Citronellol	23.6 ± 1.9	8.2 ± 3.1	nd	nd	41 ± 3.2
Geraniol	44.7 ± 2.2	nd	4.2 ± 0.1	nd	1167.4 ± 101.5
(*Z*)-Linalool oxide	19.5 ± 0.4	nd	nd	nd	46.1 ± 0.8
(*E*)-Linalool oxide	31.3 ± 1.8	nd	nd	3 ± 0.1	50.6 ± 14.7
Epoxy-linalool	10.2 ± 2.7	5.4 ± 0.3	nd	nd	5.8 ± 1.4
Citral	7 ± 1.9	3.9 ± 0.1	nd	nd	25.1 ± 3
Thymol	nd	nd	nd	108.8 ± 23.3	nd
Eucalyptol	nd	nd	24.6 ± 2.6	nd	nd
Geranic acid	174.2 ± 28.6	nd	37.8 ± 3.8	7.7 ± 1.1	273.5 ± 3.5
Farnesol	5 ± 0.6	11.9 ± 1.4	nd	nd	nd
Acetoin	11.2 ± 0.8	146,4 ± 7,3	31.4 ± 6.9	7.5 ± 3.8	7.9 ± 2.3
Acetophenone	3.6 ± 0.3	1.5 ± 0.3	1.8 ± 0.1	41.9 ± 2	2.1 ± 0.2
Methoxyacetophenone	95.6 ± 19.1	344.8 ± 134	19.5 ± 11.3	108.8 ± 23.3	98.4 ± 28.2
γ-Butyrolactone	5.4 ± 0.7	8.6 ± 3.3	2 ± 0.6	29.3 ± 2.4	11.2 ± 2.5
Benzaldehyde	0.8 ± 0	1 ± 0.3	5.4 ± 0.5	1 ± 0.1	0.8 ± 0
Phenylacetaldehyde	nd	nd	nd	nd	1.3 ± 0.5
2-Phenylethanol	0.1 ± 0	0.5 ± 0	0.9 ± 0.1	0.1 ± 0	0.1 ± 0
Benzilic acid	7.6 ± 0.3	6.3 ± 0.3	25.4 ± 12.3	2.7 ± 0.5	11.7 ± 0.8
(*E*)-Cinnamic acid	0.8 ± 0.2	70.2 ± 20.4	39.5 ± 8.2	nd	37.1 ± 17.3
Benzoic acid	2.7 ± 0.3	53.1 ± 12.3	31.9 ± 4.1	42.9 ± 5.4	30.4 ± 11
4-Vinylphenol	19.9 ± 2.4	42 ± 11.6	4.3 ± 0.6	63.7 ± 2.5	76.8 ± 7.2
Vanillin	33.2 ± 3	39.8 ± 9	6.2 ± 0.6	24.7 ± 2	20.6 ± 3.2
Phenylacetic acid	nd	nd	nd	nd	10.5 ± 1.6
Homovanillic acid	273.2 ± 18.5	51.1 ± 24.2	5.1 ± 0.6	22.2 ± 7.1	54 ± 6.9
Furaneol	10.4 ± 0.4	nd	461.4 ± 0.6	106.1 ± 3.7	nd

**Table 3:** Results obtained from the olfactometric analysis of Guzun grapes. The compounds are ordered according to the modified frequency in percentage (MF%) in a decreasing way. Initial retention time (RTI) and final retention time (RTF) of the detected odorants. Odor descriptors from [28,29]; http://www.flavornet.org and http://www.thegoodscentscompany.com (accessed on 1 April 2024).

Compounds	RT	RT_F_	MF%	Odour Descriptor by Assessors	Odour Descriptor by Literature
Eugenol	42.85	43.17	91.3	Spicy, medicinal	Clove, spices, cinnamon, honey, woody, ham, bacon
2-Phenylethanol	38.13	38.31	89.4	Rose, floral	Sweet, floral, rose, bready, honey, aromatic
Geraniol	36.74	36.97	81.6	Rose, lavander, lemon, fruity	Citric, orange, floral, roses, geranium, citronella, fruity
Phenylacetic acid	48.50	48.85	81.6	Honey, sweet	Honey, flower, rose, chocolate, tobacco, powdery, animal
Furaneol	40.04	40.30	75.3	Candy, cotton candy, caramel	Candy, sweet, candyfloss, caramellic
Vanillin	48.95	49.17	74.8	Vanillin, bakery	Vanilla, custard, sweet, creamy, phenolic
4-Ethylguaiacol	39.11	39.45	73.0	Rubber, spicy, vegetable	Clove, phenolic, spice, medicinal, woody, vanilla
Nonanoic acid	42.49	42.73	73.0	Green, cheese	Nutty, green, fat, waxy, cheesy, dairy
Hexanol	25.64	25.31	70.7	Vegetal, leaf	Green, cut grass, herbaceous, flower, fruity, ethereal, oil
Isovaleric acid	33.12	33.28	68.3	Cheese	Foot, cheese, perspiration, rancid, fruity
5-Ethoxydihydro-2(3H)-furanone	36.18	36.55	65.8	Pancake, cooked, dried fruit	
Guaiacol	37.13	37.42	65.8	Medicinal, spicy, smoke	Medicinal, spiced, smoke, sweet
2-Methoxy-3-sec-butyl pyrazine	29.63	29.82	63.2	Green pepper, peas	Pepper, earthy
Linalool	30.25	30.39	63.2	Orange blossom, floral, rose, white flower	Citrus, orange, grape, floral, lavender, rose, waxy
3-Mercaptohexyl acetate	34.38	34.58	63.2	Boxwood, peppermint	Boxwood, basil, tropical fruit, passion fruit, black currant, sulfurous, roasted meat
Rotundone	44.27	44.37	63.2	Pepper	Peppery, woody
4-Mercapto-4-methyl-2-pentanone	25.80	25.92	60.6	Boxwood, Sauvignon	Boxwood, green, urine
Octanoic acid	40.92	41.04	60.6	Vegetable, unpleasant	Rancid, perspiration, plastic, cheese
Methoxybenzaldehyde	41.56	41.80	60.6	Spicy	
Sotolon	42.33	42.47	60.6	Curry, liquorice	Liquorice, toasted, curry, spice, cotton candy, maple
Decanoic acid	46.96	47.13	60.6	Rancid, wax, Marseille soap	Rancid, perspiration, fat
Decanol	35.42	35.75	57.7	Bug, oily, fried	Fat, waxy, oily, floral, rose, citrus
2-Hexenoic acid	41.93	42.07	57.7	Mint, spicy,	Fatty, rancid
Ethyl hexadecanoate	43.83	44.03	57.7	Spicy, fruity, cheese	Waxy, fruity, creamy, milky, balsamic
Citronellol	34.19	34.38	50.0	Citrus, lemongrass, lemon	Floral, rose, sweet, citrus, green, fatty
2,4-Heptadienal	28.40	28.88	49.4	Cucumber, bug	Rancid, fat, fried, cucumber, citrus, melon, fruity, spicy
2-Ethyl-3-methylpyrazine	27.37	27.47	48.3	Peas, green pepper	Green pea, nutty, peanut, musty, earthy, bready, roasted, oil, potato, cereal, coffee, cocoa
Furfural	32.13	32.48	48.3	Fried, pancake	Sweet wood, nut, almond, bread, caramellic, phenolic
Citral	34.05	34.45	48.3	Balsamic, cut grass	Lemon, lemon peel, juicy, green
Ethyl hydrocinnamate	37.53	37.73	48.3	Cinnamon	Labdanum, honey, fruity rum, hyacinth, rose
4-Vinylphenol	43.38	43.49	48.3	Medicinal, rubber	Almond shell, chemical, phenolic, medicinal, musty
Methoxyacetophenone	46.73	46.94	44.7	Caramel, raspberry	
Lauric Acid	47.46	47.69	44.7	Wax	Metal
2-Acetyl-3-methylpyrazine	31.57	31.70	44.7	Cucumber, tzatziki	Hazelnut, roasted, potato, baked, vegetable, cereal, caramel
1-Octen-3-one	22.81	22.99	44.7	Mushroom	Mushroom, earthy, vegetative, creamy, fishy
Hexanoic acid	37.24	37.59	44.7	Medicine, peanuts	Rancid, cheese, fatty, sweat
Ethyl octanoate	27.52	27.58	40.8	Fruity, pineapple, floral	Sweet, floral, fruity, banana, pear, pineapple, brandy, fat, waxy, musty, creamy, dairy
Dimethyl-7-octen-2-ol	31.72	31.91	40.8	Vegetal, bark	Herbal, lime, bergamot, tropical fruit, soapy, floral
Dodecanal	34.66	34.84	40.8	Aromatic vegetable, floral	Metallic, sea, soapy, waxy, citrus, orange, mandarin, woody
Benzyl alcohol	37.84	38.02	40.8	Rose, flower	Roasted, toasted, sweet, fruity, flower, rose, grass, sweet, chemical, phenolic, balsamic, almond
Maltol	38.87	39.04	40.8	Fruity, cooked	Sweet, caramellic, cotton candy, jammy, fruity, burnt, bready

**Table 4:** Results obtained from the olfactometric analysis of the Melona grape. The compounds are ordered according to the modified frequency in percentage (MF%) in a decreasing way. Initial retention time (RTI) and final retention time (RTF) of the detected odorants. Odor descriptors from [28,29]; http://www.flavornet.org and http://www.thegoodscentscompany.com (accessed on 1 April 2024).

Compounds	RT_I_	RT_F_	MF%	Odour Descriptor by Assessors	Odour Descriptor by Literature
Furaneol	40.02	40.27	87.6	Candy, candy cotton, jam	Candy, sweet, candyfloss, caramellic
n.i.	36.71	37.13	85.6	Rose, floral	
Sotolon	42.24	42.42	81.6	Curry, liquorice	Liquorice, toasted, curry, spice, cotton candy, maple
Eugenol	42.80	43.02	81.6	Spicy, medicinal	Clove, spices, cinnamon, honey, woody, ham, bacon
Phenylacetic acid	48.40	48.83	79.6	Honey, sweet	Honey, flower, rose, chocolate, tobacco, powdery, animal
Nonanoic acid	42.53	42.68	77.5	Green, cheese	Nutty, green, fat, waxy, cheesy, dairy
2-Phenylethanol	38.06	38.30	75.3	Rose, floral	Sweet, floral, rose, bready, honey, aromatic
4-Ethylguaiacol	39.10	39.30	73.0	Spicy, vanilla, vegetable, phenolic, coconut	Clove, phenolic, spice, medicinal, woody, vanilla
n.i.	40.32	40.54	70.7	Floral, fruity	
Decanoic acid	43.42	43.56	70.7	Rancid, wax, Marseille soap	Rancid, perspiration, fat
Hexanoic acid	36.30	36.55	68.3	Medicine, peanuts	Rancid, cheese, fatty, sweat
2-Hexenoic acid	41.86	42.20	68.3	Mint, spicy	Fatty, rancid
n.i.	35.90	36.25	67.7	Meat, tosted, smoky	
Acetophenone	41.43	41.58	67.7	Wood, melon	Must, flower, spicy, almond, nuts, fruity, cherry, strawberry
Octanoic acid	40.86	41.09	65.8	Vegetable, unpleasant	Rancid, perspiration, plastic, cheese
Methoxybenzaldehyde	41.68	41.84	65.8	Spicy	
Ethyl octanoate	27.41	27.71	63.2	Fruity, pineapple, floral	Sweet, floral, fruity, banana, pear, pineapple, brandy, fat, waxy, musty, creamy, dairy
Decanol	35.43	35.65	63.2	Bug, oily, fried	Fat, waxy, oily, floral, rose, citrus
2-Decenal	29.56	29.86	60.6	Violet, melon	Orange, floral, rose, green
Phenylacetaldehyde	31.69	31.86	60.6	Floral	Flowery, rose, green, honey, powdery, chocolate, earthy
Ethyl hydrocinnamate	37.29	37.59	60.6	Cinnamon	Labdanum, honey, fruity rum, hyacinth, rose
Methyl salicylate	38.42	38.70	60.6	Balsamic, citrus	Peppermint, green, sweet, phenolic, camphoreous
Rotundone	44.21	44.31	60.6	Black pepper	Peppery, woody
Phenol	32.30	32.43	57.7	Cheese	Phenolic, plastic, rubber
Ethyl hexadecanoate	43.89	44.02	57.7	Spicy, fruity, cheese	Waxy, fruity, creamy, milky, balsamic
Lauric Acid	47.32	47.60	57.7	Wax	Metal
2,4-Heptadienal	28.22	28.47	54.8	Cucumber, bug	Rancid, fat, fried, cucumber, citrus, melon, fruity, spicy
Linalool	30.06	30.32	54.8	Orange blossom, floral, rose, white flower	Citrus, orange, grape, floral, lavender, rose, waxy
α-Terpineol	30.27	30.45	54.8	Floral, balsamic	Floral, lilac, sweet, anise, mint, green, oil
Isovaleric acid	33.15	33.28	54.8	Cheese, foot	Foot, cheese, perspiration, rancid, fruity
Benzyl alcohol	37.77	37.93	54.8	Rose, flower	Roasted, toasted, sweet, fruity, flower, rose, grass, sweet, chemical, phenolic, balsamic, almond
Isovaleric acid	28.55	28.86	51.6	Cheese, foot	Foot, cheese, perspiration, rancid, fruity
4-Mercapto-4-methyl-2-pentanone	25.71	25.86	48.3	Green pepper, rocket	Boxwood, green, urine
Furfural	32.83	33.02	48.3	Burny, oil, toasted	Sweet wood, nut, almond, bread, caramellic, phenolic
Ethyl decanoate	28.87	28.95	44.7	Green apple, blackberry	Fruity, grape, apple, nutty, sweet, waxy, oily, wine, yeast
Maltol	38.83	39.07	44.7	Candy cotton, caramel	Sweet, caramellic, cotton candy, jammy, fruity, burnt, bready
Vanillin	49.09	49.48	44.7	Vanilla, bakery	Vanilla, custard, sweet, creamy, phenolic
Isoamyl alcohol	15.49	15.69	40.8	Wet rag	Foot, solvent, fusel, alcoholic, pungent, etherial, cognac, brandy, wine, fruity, banana, molasses
2-Methoxy-3-sec-butyl pyrazine	29.99	30.07	40.8	Pepper	Pepper, earthy
Dodecanal	34.50	34.67	40.8	Mint, aromatic vegetable, floral, waxy	Metallic, sea, soapy, waxy, citrus, orange, mandarin, woody
(*E*)-Hexenoic acid	39.80	39.97	40.8	Herbaceous	Must, fat, cheesy, caramel

**Table 5:** Results obtained from the olfactometric analysis of Cotton Candy grape. The compounds are ordered according to the modified frequency in percentage (MF%) in a decreasing way. Initial retention time (RTI) and final retention time (RTF) of the detected odorants. Odor descriptors from [28,29]; http://www.flavornet.org and http://www.thegoodscentscompany.com (accessed on 1 April 2024).

Compounds	RT_I_	RT_F_	MF%	Odour Descriptor by Assessors	Odour Descriptor by Literature
Hexanol	25.59	26.03	93.8	Vegetable, leaf	Green, cut grass, herbaceous, flower, fruity, etherial, oil
Sotolon	42.68	42.94	93.8	Curry, liquorice	Liquorice, toasted, curry, spice, cotton candy, maple
Eugenol	42.89	43.13	93.1	Spicy, cloves	Clove, spices, cinnamon, honey, woody, ham, bacon
4-Ethylphenol	42.36	42.55	85.6	Horse, phenolic	Phenolic, leather, smoke
Decanoic acid	43.38	43.63	82.5	Wax, Maeseille soap	Rancid, perspiration, fat
2-Phenylethanol	36.77	37.03	81.1	Rose, floral	Sweet, floral, rose, bready, honey, aromatic
2-Dodecenal	29.70	29.98	77.5	Watemelon, bug	Fat, waxy, herbal, cilantro, citrus peel
Butyric acid	33.19	33.32	75.3	Cheese	Cheesy, caprylic, butter, fruity
4-Vinylphenol	43.74	43.92	75.3	Spicy, medicinal	Almond shell, chemical, phenolic, medicinal, musty
Methyl salicylate	38.22	38.50	74.5	Balsamic, citrus	Peppermint, green, sweet, phenolic, camphoreous
Furaneol	39.99	40.33	74.5	Caramel	Candy, sweet, candyfloss, caramellic
1-Octen-3-one	22.68	22.98	73.0	Mushroom	Mushroom, earthy, vegetative, creamy, fishy
Rotundone	44.18	44.35	73.0	Black pepper	Peppery, woody
Phenylacetic acid	48.46	48.69	73.0	Honey, sweet	Honey, flower, rose, chocolate, tobacco, powdery, animal
4-Ethylguaiacol	38.90	39.11	72.6	Rubber, phenolic, spicy	Clove, phenolic, spice, medicinal, woody, vanilla
Decanal	34.11	34.38	70.7	Bug, oregan	Sweet, waxy, citrus rind, floral
Octanoic acid	40.90	41.00	70.7	Unpleasant	Rancid, perspiration, plastic, cheese
Vanillin	48.94	49.06	70.7	Vanilla, sweet	Vanilla, custard, sweet, creamy, phenolic
Ethyl octanoate	27.52	27.60	68.3	Fruity, pineapple, floral	Sweet, floral, fruity, banana, pear, pineapple, brandy, fat, waxy, musty, creamy, dairy
Geraniol	36.05	36.38	67.1	Rose, citrus, floral, white flower	Citric, orange, floral, roses, geranium, citronella, fruity
2-Acetyl-3-methylpyrazine	30.00	30.18	65.8	Potatoes, vegetable	Hazelnut, roasted, potato, baked, vegetable, cereal, caramel
Methoxybenzaldehyde	41.87	42.09	63.2	Spicy	
Ethyl hydrocinnamate	37.12	37.37	60.6	Cinnamon	Labdanum, honey, fruity rum, hyacinth, rose
Methoxyacetophenone	41.38	41.61	59.2	Caramel, raspberry	
Linalool	30.43	30.56	57.7	Orange blossom, floral, rose, white flower	Citrus, orange, grape, floral, lavender, rose, waxy
Isovaleric acid	32.54	32.79	57.7	Cheese	Foot, cheese, perspiration, rancid, fruity
(*E*)-Cinnamaldehyde	47.39	47.59	57.0	Cinnamon, spicy	Cinnamon, spicy, paint
Furfural	32.16	32.32	54.8	Baked potatoes, candy	Sweet wood, nut, almond, bread, caramellic, phenolic
Guaiacol	36.44	36.62	54.8	Phenolic, spicy	Medicinal, spiced, smoke, sweet
Hexanoic acid	37.63	37.80	54.8	Fruity, wax	Rancid, cheese, fatty, sweat
Eucalyptol	17.73	17.92	51.6	Balsamic, mint, sage	Camphoric, mint, sweet, eucalyptus, camphoraceous
2,4-Heptadienal	28.03	28.28	51.6	Bug, cucumber	Rancid, fat, fried, cucumber, citrus, melon, fruity, spicy
Decanol	33.51	34.03	51.6	Oily, peanuts	Fat, waxy, oily, floral, rose, citrus
Ethyl decanoate	27.83	28.01	48.3	Green apple, blackberry	Fruity, grape, apple, nutty, sweet, waxy, oily, wine, yeast
Benzaldehyde	28.62	28.92	48.3	Cake, cookies	Sweet, fruity, roasted, almond, nutty, fragant, burnt sugar, powdery, cherry, floral
3(2H)-Furanone	31.12	31.30	48.3	Toasted, smoky	
2-Ethyl-3-methylpyrazine	27.15	27.34	47.1	Green pea, vegetable	Green pea, nutty, peanut, musty, earthy, bready, roasted, oil, potato, cereal, coffee, cocoa
Lauric Acid	46.21	46.42	40.8	Wax	Metal

**Table 6:** Results obtained from the olfactometric analysis of the IVC SB1 grape. The compounds are ordered according to the modified frequency in percentage (MF%) in a decreasing way. Initial retention time (RTI) and final retention time (RTF) of the detected odorants. Odor descriptors from [28,29]; http://www.flavornet.org and http://www.thegoodscentscompany.com (accessed on 1 April 2024).

Compounds	RT_I_	RT_F_	MF%	Odour Descriptor by Assessors	Odour Descriptor by Literature
Furaneol	39.99	40.18	87.8	Smoky, candyfloss	Candy, sweet, candyfloss, caramellic
(*E*)-2-Hexenoic acid	40.94	41.06	83.7	Floral, fruity	Must, fat, cheesy, caramel
Ethyl hydrocinnamate	37.18	37.36	77.5	Cinnamon, spicy	Roasted, toasted, sweet, fruity, flower, rose, grass, sweet, chemical, phenolic, balsamic, almond
Phenylacetic acid	47.74	47.98	77.5	Honey, vanilla sweet	Honey, flower, rose, chocolate, tobacco, powdery, animal
2-Methoxy-3-sec-butyl pyrazine	28.50	28.76	71.2	Green pepper	Pepper, earthy
2-Phenylethanol	38.22	38.35	70.7	Rose, floral	Sweet, floral, rose, bready, honey, aromatic
Maltol	38.82	39.02	70.7	Cinnamon, spicy, cooked	Sweet, caramellic, cotton candy, jammy, fruity, burnt, bready
Nonanoic acid	42.54	42.77	70.7	Green, cheese	Nutty, green, fat, waxy, cheesy, dairy
Eugenol	42.87	43.14	68.3	Spicy, cloves, vanilla	Clove, spices, cinnamon, honey, woody, ham, bacon
Hexanoic acid	36.74	37.04	65.8	Fruity, wax	Rancid, cheese, fatty, sweat
Decanol	35.39	35.57	63.2	Oily, peanuts	Fat, waxy, oily, floral, rose, citrus
2-Decenal	26.27	26.37	60.6	Grass, pea,	Orange, floral, rose, green
Geraniol	36.21	36.34	60.6	Rose, citrus, floral, white flower	Citric, orange, floral, roses, geranium, citronella, fruity
Lauric Acid	47.30	47.55	60.6	Candy	Metal
4-Vinylphenol	43.20	43.42	59.2	Leather. medicinal	Almond shell, chemical, phenolic, medicinal, musty
Linalool	30.12	30.35	57.7	Orange blossom, floral, rose, white flower	Citrus, orange, grape, floral, lavender, rose, waxy
Vanillin	48.65	48.87	57.7	Vanilla, sweet	Vanilla, custard, sweet, creamy, phenolic
(*E*)-Hexenoic acid	38.02	38.17	54.8	Rubber, vegetables	
2-Dodecenal	29.49	29.68	51.6	Herbaceous, waxy	Fat, waxy, herbal, cilantro, citrus peel
Benzyl alcohol	30.47	30.68	51.6	Floral, fruity, pleasant	Roasted, toasted, sweet, fruity, flower, rose, grass, sweet, chemical, phenolic, balsamic, almond
Isovaleric acid	33.33	33.46	51.6	Cheese, foot	Foot, cheese, perspiration, rancid, fruity
Methylbutyric acid	37.49	37.64	51.6	Wet rag, closed wardrobe	Cheese, sweat
Geranic acid	44.76	44.90	51.6	Rose	Green, woody, sweet
Isobutyl acetate	8.64	8.91	48.3	Fruity, blackberry	Fruit, apple, banana, ethereal
2-Ethyl-3-methylpyrazine	26.82	27.19	48.3	Green pepper	Green pea, nutty, peanut, musty, earthy, bready, roasted, oil, potato, cereal, coffee, cocoa
Ethyl decanoate	29.18	29.29	48.3	Floral, strawberry	Fruity, grape, apple, nutty, sweet, waxy, oily, wine, yeast
Methyl salicylate	38.43	38.59	48.3	Balsamic, citrus	Peppermint, green, sweet, phenolic, camphoreous
Furfural	32.64	32.74	44.7	Burny oil, toasted	Sweet wood, nut, almond, bread, caramellic, phenolic
Octanoic acid	41.69	41.76	44.7	Unpleasant	Rancid, perspiration, plastic, cheese
Ethyl hexadecanoate	44.04	44.15	44.7	Strawberry, fruity	Waxy, fruity, creamy, milky, balsamic
Rotundone	44.24	44.39	44.7	Black pepper	Peppery, woody
(*E*)-2-Hexenal	19.39	19.62	40.8	Grass	Grass, green, herbaceous, leaf, fruity, spicy
Dihydro-3-methyl-2(3H)-furanone	19.93	19.95	40.8	Caramel	
1-Octen-3-one	22.95	23.03	40.8	Mushroom	Mushroom, earthy, vegetative, creamy, fishy
(*Z*)-Linalool oxide	24.42	24.70	40.8	Floral, mint, sage	Floral, green
Ethyl octanoate	27.92	27.94	40.8	Fruity, pineapple, floral	Sweet, floral, fruity, banana, pear, pineapple, brandy, fat, waxy, musty, creamy, dairy
4-Ethylphenol	43.83	44.05	40.8	Horse, medicinal	Phenolic, leather, smoke
Benzoic acid	45.84	46.01	40.8	Spicy, aromatic herbs	Urine
2-Heptenal	23.61	23.72	36.5	Bug	Green, fatty

**Table 7:** Results obtained from the olfactometric analysis of the IVC SA3 grape. The compounds are ordered according to the modified frequency in percentage (MF%) in a decreasing way. Initial retention time (RTI) and final retention time (RTF) of the detected odorants. Odor descriptors from [28,29]; http://www.flavornet.org and http://www.thegoodscentscompany.com (accessed on 1 April 2024).

Compounds	RT_I_	RT_F_	MF%	Odour Descriptor by Assessors	Odour Descriptor by Literature
Rotundone	43.79	43.95	83.7	Spicy, black pepper	Peppery, woody
Furaneol	39.36	39.56	77.5	Cookies, cotton candy, caramel	Candy, sweet, candyfloss, caramellic
Eugenol	41.98	42.20	77.5	Cloves, spicy, phenolic	Clove, spices, cinnamon, honey, woody, ham, bacon
Sotolon	42.44	42.64	77.5	Curry, liquorice	Liquorice, toasted, curry, spice, cotton candy, maple
Vanillin	47.95	48.16	77.5	Vanilla	Vanilla, custard, sweet, creamy, phenolic
2,4-Heptadienal	27.47	27.84	70.7	Bug, vegetable, solvent	Rancid, fat, fried, cucumber, citrus, melon, fruity, spicy
Linalool	29.51	29.84	70.7	Orange blossom, floral, rose, white flower	Citrus, orange, grape, floral, lavender, rose, waxy
Eugenol	36.71	36.99	70.7	Spicy, cloves, vanilla	Clove, spices, cinnamon, honey, woody, ham, bacon
4-Ethylguaiacol	38.66	38.79	70.7	Medicinal, spicy	Clove, phenolic, spice, medicinal, woody, vanilla
Hexanol	26.68	26.90	65.8	Peas	Green, cut grass, herbaceous, flower, fruity, etherial, oil
Geraniol	35.87	36.15	65.8	Lemon, rose, floral	Citric, orange, floral, roses, geranium, citronella, fruity
Citral	33.82	34.02	63.2	Mint, lemon	Lemon, lemon peel, juicy, green
(*E*)-2-Hexenoic acid	39.04	39.25	63.2	Peas	Must, fat, cheesy, caramel
Methoxyacetophenone	40.43	40.63	63.2	Strawberry candy	
(*Z*)-3-Hexenol	25.15	25.39	57.7	Leaf, aromatic herbs	Cut grass, herbaceous, foliage, bitter, grassy, melon rind
Citronellol	34.22	34.48	57.7	Rose, peach, dried fruit	Floral, rose, sweet, citrus, green, fatty
Guaiacol	37.22	37.31	57.7	Smoky, balsamic	Medicinal, spiced, smoke, sweet
2-Methoxy-3-sec-butyl pyrazine	29.06	29.35	54.8	Green pepper, peas	Pepper, earthy
Furfural	32.99	33.23	54.8	Peanuts, pancakes	Sweet wood, nut, almond, bread, caramellic, phenolic
α-Terpineol	33.55	33.74	51.6	Mint	Floral, lilac, sweet, anise, mint, green, oil
Benzyl alcohol	37.67	37.81	51.6	Floral, rose, vegetable	Roasted, toasted, sweet, fruity, flower, rose, grass, sweet, chemical, phenolic, balsamic, almond
2-Phenylethanol	38.19	38.35	51.6	Rose, floral	Sweet, floral, rose, bready, honey, aromatic
Nonanoic acid	43.19	43.40	51.6	Vanilla, green vegetable	Nutty, green, fat, waxy, cheesy, dairy
β-Damascenone	34.96	35.08	48.3	Floral, peach	Sweet, floral, rose, fruity, plum, berry, apple, baked apple, boiled apple, compote, honey, woody, earthy, tobacco
5-Ethoxydihydro-2(3H)-furanone	35.73	35.96	48.3	Fried potatoes, tobacco	
Hexanoic acid	37.54	37.67	48.3	Smoky, rancid	Rancid, cheese, fatty, sweat
Decanoic acid	45.24	45.42	48.3	Wax, Marseille soap	Rancid, perspiration, fat
Lauric Acid	45.79	45.97	48.3	Wax	Metal
(*E*)-Cinnamaldehyde	46.86	47.06	48.3	Cinnamon	Cinnamon, spicy, paint
2-Acetyl-3-methylpyrazine	30.57	30.74	44.7	Vegetable, salad	Hazelnut, roasted, potato, baked, vegetable, cereal, caramel
Maltol	38.42	38.56	44.7	Aromatic vegetable, caramel	Sweet, caramellic, cotton candy, jammy, fruity, burnt, bready
2-Ethyl-3-methylpyrazine	29.38	29.49	40.8	Green pepper, peas, wax	Green pea, nutty, peanut, musty, earthy, bready, roasted, oil, potato, cereal, coffee, cocoa
Hotrienol	31.10	31.30	40.8	Sweet	Fresh, floral, hyacinth, fruity
Hexanoic acid	36.25	36.50	40.8	Floral, fruiy	Rancid, cheese, fatty, sweat

## Data Availability

Data are contained within the article.
